# The aptamer BT200 blocks von Willebrand factor and platelet function in blood of stroke patients

**DOI:** 10.1038/s41598-021-82747-7

**Published:** 2021-02-04

**Authors:** Katarina D. Kovacevic, Stefan Greisenegger, Agnes Langer, Georg Gelbenegger, Nina Buchtele, Ingrid Pabinger, Karin Petroczi, Shuhao Zhu, James C. Gilbert, Bernd Jilma

**Affiliations:** 1 Department of Clinical Pharmacology, Medical University of Vienna, Währinger Gürtel 18-20, 1090 Vienna, Austria; 2 Department of Neurology, Medical University of Vienna, Vienna, Austria; 3 Department of Internal Medicine I, Medical University of Vienna, Vienna, Austria; 4 Division of Hematology, Department of Internal Medicine I, Medical University of Vienna, Vienna, Austria; 5 Guardian Therapeutics, Lexington, MA USA

**Keywords:** Cerebrovascular disorders, Stroke

## Abstract

The effect of conventional anti-platelet agents is limited in secondary stroke prevention, and their effects are blunted under high shear stress in the presence of increased levels of circulating von Willebrand factor (VWF). VWF is critically involved in thrombus formation at sites of stenotic extracranial/intracranial arteries. A third generation anti-VWF aptamer (BT200) has been generated which could be useful for secondary stroke prevention. To characterize the effects of BT200 in blood of patients with large artery atherosclerosis stroke (LAA). Blood samples were obtained from 33 patients with acute stroke or transient ischemic attack to measure inhibition of VWF activity and VWF-dependent platelet function. Patients who received clopidogrel or dual antiplatelet therapy did not differ in VWF dependent platelet function tests from aspirin treated patients. Of 18 patients receiving clopidogrel with or without aspirin, only 3 had a prolonged collagen adenosine diphosphate closure time, and none of the patients had ristocetin induced aggregation in the target range. BT200 concentration-dependently reduced median VWF activity from 178 to < 3%, ristocetin induced platelet aggregation from 40U to < 10U and prolonged collagen adenosine diphosphate closure times from 93 s to > 300 s. Baseline VWF activity correlated (r = 0.86, p < 0.001) with concentrations needed to reduce VWF activity to < 20% of normal, indicating that BT200 acts in a target concentration-dependent manner. Together with a long half-life supporting once weekly administration, the safety and tolerability observed in an ongoing phase I trial, and the existence of a reversal agent, BT200 is an interesting drug candidate.

## Introduction

The burden of stroke on our healthcare delivery system continues to grow as our population ages and the incidence and prevalence of stroke rises. In the US, each year ~ 795,000 people experience a new or recurrent stroke. Between 2015 and 2035, total direct medical stroke-related costs are projected to more than double, from $36.7 billion to $94.3 billion^1^. Globally, the problem is even larger. In China, for example, stroke is now the leading cause of death despite advances in underlying risk factor management. Furthermore, stroke event rates and stroke-related mortality rates do not capture the full impact on society. Since a non-fatal stroke is often disabling, stroke-related Disability Adjusted Life Years lost is on par with that related to ischemic heart disease^2^.

Many of the antiplatelet drugs are effective and useful in coronary disease but when it comes to the cerebral vascular diseases or stroke, heparin^3,4^ and antiplatelet drugs, GPIIb/IIIa inhibitors like abciximab^5^ or tirofiban^6,7^, P2Y12 blockers such as prasugrel^8^ or the thrombin-receptor antagonist vorapaxar^9^ cause treatment-induce bleedings and they are contraindicated in stroke. Current secondary prevention of non-cardioembolic stroke consists of aspirin or clopidogrel alone, or aspirin in combination with dipyridamole or clopidogrel in some circumstances, and there is an obvious need for new and improved anti-thrombotic therapy in this field.

BT200 is a third-generation anti- Von Willebrand factor (VWF) aptamer^10^ which inhibits the VWF A1 domain and therefore inhibits the VWF-GPIb axis. VWF is a large glycoprotein and plays a major role in thrombus formation^11^: It forms large functional multimers that can elongate to long strings that capture platelets in cases of moderate to high shear forces^12–15^. Because VWF is a key factor for the platelet plug formation at high shear rates, other platelet inhibitors such as GPIIb/IIIa (abciximab), P2Y12 (clopidogrel, ticagrelor, prasugrel or cangrelor) or cyclooxygenase-1 (aspirin) are less potent when VWF levels are increased^16–18^. As VWF is a driver^19^ and an independent predictor of stroke recurrence^20^ and mortality^21,22^ and there is a need for improved stroke therapy, we wanted to explore the concentration-effect curves of BT200 in the blood of stroke patients. We hypothesized that BT200 concentration-dependently inhibits platelet function ex vivo and that concentrations needed to inhibit VWF will be target concentration-dependent.

## Methods

This was a non-interventional study performed by using in vitro assays on blood samples collected from 33 patients suffering from stroke due to Large Artery Atherosclerosis (LAA), who provided written informed consent after Ethics Committee approval. Inclusion criteria for patients were that they were older than 18 years, able to give informed consent; they had an established diagnosis of extra- or intra-cranial, large vessel cerebrovascular disease and history of TIA/stroke or a carotid revascularization procedure. Patients were excluded if they were currently treated with unfractionated heparin, warfarin, or a Novel Oral Anti-Coagulant (e.g. rivaroxaban, apixaban, dabigatran, etc.).

The study protocol was designed in accordance with the Declaration of Helsinki and approved by the Ethics Committee of the Medical University of Vienna. Patients were recruited at the Medical University of Vienna and Vienna General Hospital department of Neurology. Blood samples were collected from an antecubital vein via a butterfly venipuncture device and transferred to the appropriate blood collection container either to be analyzed immediately or to be centrifuged for subsequent analysis.

All assays were conducted in the laboratory of the Department of Clinical Pharmacology. In order to characterize the concentration-effect relationship for BT200 in a set of different assays thought to be specifically related to VWF function, 4 assays that are specifically designed to measure the binding of VWF via its A1 domain to platelet GPIb were performed. The first analysis was done with Platelet function analyzer PFA-100 (Dade Behring)—an approved medical device normally used for the diagnosis of deficiency of VWF in whole-blood, a functional assay that quantifies the rate at which a platelet “plug” can form under shear stress in a VWF-dependent process. We have measured collagen adenosine diphosphate (CADP-CT) induced closure time^23^. The second analysis was done with Multiple Electrode Aggregometry (MEA) (Multiplate). Ristocetin was used as an agent to measure ristocetin induced aggregation time^24^, this test is sensitive to congenital as well as acquired VWF deficiency^25^. The third analysis was VWF:RCo, it is a standard clinical laboratory assay that quantifies the activity of VWF in the patient’s sample via agglutination of donor platelets, using added ristocetin to circumvent the physiologic requirement for the shear force to activate VWF^26^. The fourth analysis was REAADS VWF activity, it is an enzyme immunoassay that specifically recognizes the portion of VWF which binds to platelets (A1 domain)^27^. For each of these assays, a series of spiked-in concentrations of BT200 from 0.1 to 15 μg/mL were tested.

We used ROTEM thromboelastometry (TemInnovations) to rule out an undue interference with the coagulation system, it measures clotting times (CT and CFT). No activator was used but samples were only recalcified^28^.

Patients with average VWF plasma levels of 24% that of normal had a 35–67% reduced risk for ischemic stroke as compared to controls, suggesting that partial inhibition of VWF could be protective in a stroke-prone population^29^ which is why we aimed to reach < 20% of normal VWF activity. Two-sided p-values of p < 0.05 were considered significant.

### Statistical analysis

Data were analyzed descriptively. Non-parametric tests were applied as appropriate. We used a Kruskal Wallis test and the U-test to compare the lowest concentrations that will maximally prolong CADP-CT or reduce ristocetin induced aggregation to < 20U between different groups. A Spearman ranks correlation test was applied to estimate the association between baseline VWF levels and BT200 concentrations needed to reduce VWF activity to < 20% of normal.

## Results

In total, 33 LAA patients were included in the study whose baseline demographic data and clinical characteristics are given in Table 1. Seventy-nine percent of patients suffered from stroke and 21% from transient ischemic attack and they were included on average 6 days after the event.

**Table 1:** Demographic characteristics and baseline laboratory parameters. Data are reported as mean standard deviation (SD), median interquartile range (IQR) or n (number of patients) with percentages.

	Mean ± SD, [Median ± IQR] or n (%)
	N = 33
Age (years)	72 ± 10 [73 ± 16]
Weight (kg)	75 ± 13 [77.5 ± 17]
Gender (male)	23 (70%)
**Risk factors/past medical history n (%)**	
Diabetes	13 (39)
Hypertension	23 (70)
Previous stroke	13 (39)
Smoking	18 (54.5)
Hypercholesterolemia	19 (58)
Stenosis location left	9 (27)
Stenosis location right	15 (45)
Stenosis location both sides	9 (27)
Stroke location left	11 (33)
Stroke location right	15 (45)
Transient ischemic attack	7 (21)
Stenosis and stroke not on the same side	1 (33)
**Medication n (%)**	
Aspirin	23 (70)
Clopidogrel	18 (55)
Statins	18 (55)
Proton pump inhibitors	9 (27)
Antidiabetics	11 (33)
**Laboratory data (mean ± SD) [median ± IQR]**	
Hemoglobin (g/dl)	13.1 ± 2.21 [12.95 ± 3.9]
Platelets (109/L)	271 ± 114 [250 ± 122]
Mean platelet volume (fL)	10.4 ± 1.8 [10.2 ± 1.5]
Fibrinogen (mg/dl)	429 ± 118 [404 ± 166]
Creatinine (mg/dl)	0.88 ± 0.31 [0.79 ± 0.38]
High-sensitivity C-reactive protein (mg/L)	2.0 ± 4.8 [0.39 ± 1.55]
**VWF dependent assays (mean ± SD) [median ± IQR]**	
VWF activity REAADS ELISA (%)	187 ± 75 [178 ± 75]
Ristocetin cofactor activity (%)	190 ± 75 [171 ± 114]
Ristocetin induced aggregation (U)	50 ± 36 [40 ± 30]
Collagen adenosine diphosphate closure time (s)	116 ± 74 [93 ± 43]

*VWF* Von Willebrand Factor.

**Figure 1:**
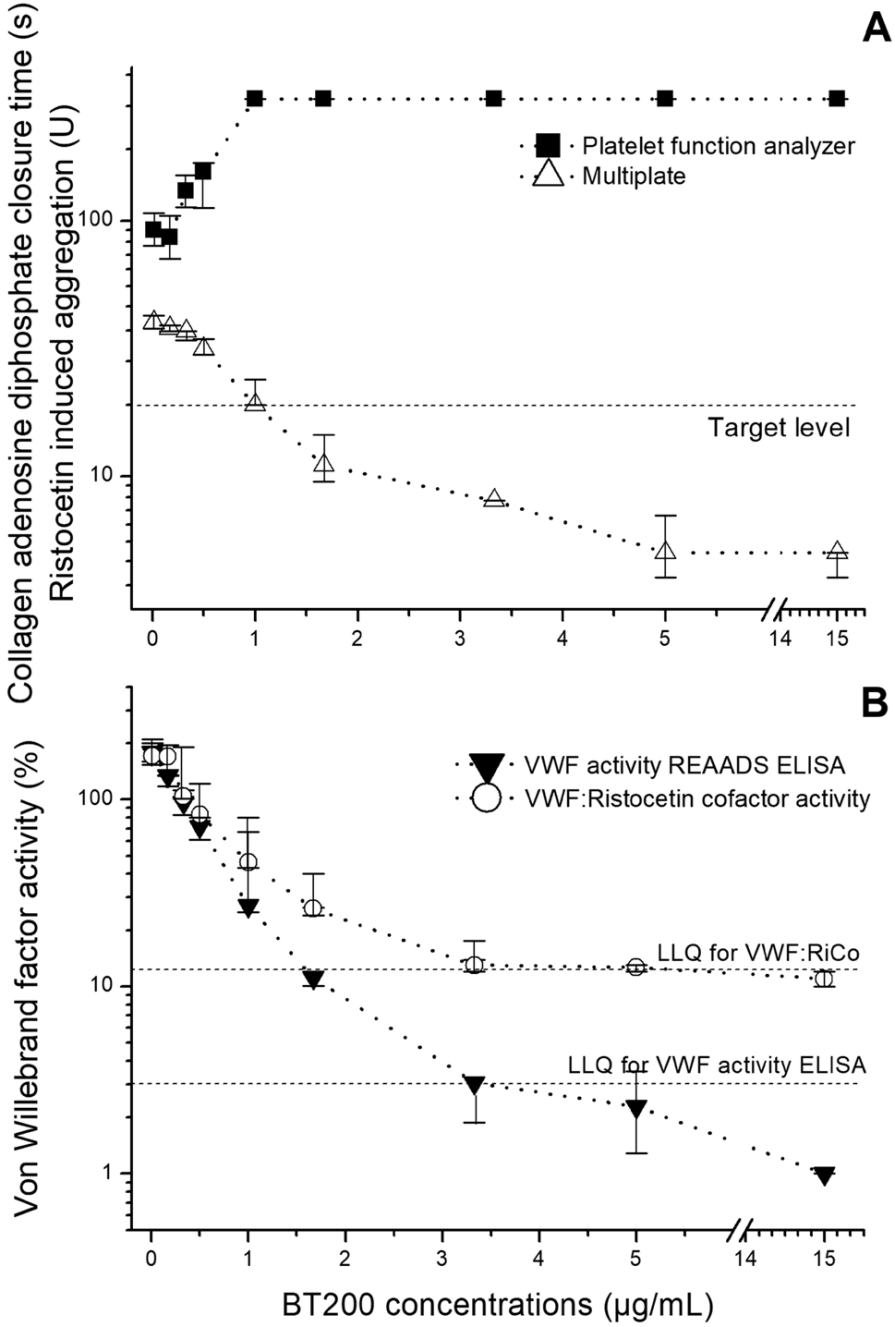
Concentration effect curves of the von Willebrand factor (VWF) inhibiting aptamer BT200 on platelet plug formation under high shear stress, ristocetin-induced whole blood aggregation (**A**), VWF activity and ristocetin cofactor activity (RCo; **B**) in patients with large artery atherosclerosis stroke or transient ischemic attacks. BT200 was spiked ex vivo into plasma or whole blood. Collagen adenosine diphosphate closure time (CADP-CT) and aggregation were measured by the platelet function analyzer PFA-100 and Multiplate. Data are presented as median values and the interquartile range.

The median VWF activity level was 178% (IQR: 103–253%) and half of the patients had elevated levels (> 180%). Average concentrations of 1.3 µg/ml BT200 were needed to suppress VWF activity to < 20% of normal in this population (Fig. 1). The median VWF:RCo was 171% (IQR: 57–285%) and an average concentration of 2.5 µg/ml BT200 was needed to suppress VWF:RCo to < 20% of normal (Fig. 1). The baseline value of CADP-CT measured with PFA was 93 s (IQR: 50–136 s) and 1 µg/ml BT200 was needed to maximally prolong the CADP-CT to > 300 s (Fig. 1). The median value of ristocetin induced aggregation was 40U (IQR: 10–79U) and 1 µg/ml BT200 was needed to inhibit aggregation to < 20U (Fig. 1).

**Figure 2:**
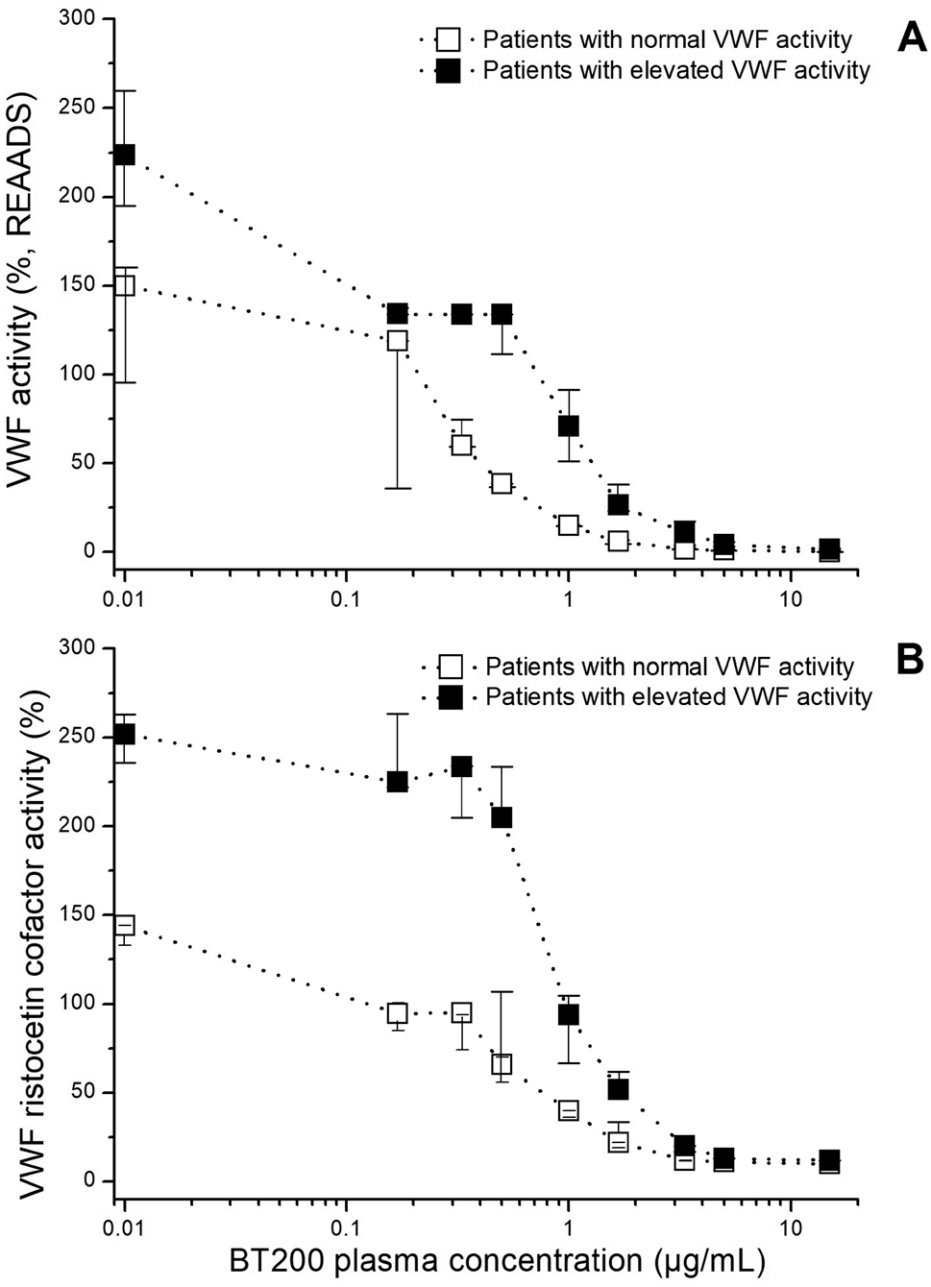
Target concentration dependent effects of BT200 on von Willebrand factor (VWF) activity (**A**) and VWF ristocetin cofactor activity (**B**) in patients with large artery atherosclerosis stroke or transient ischemic attacks when stratified into groups with normal (< 180%) or elevated (> 180%) VWF activity. Data are presented as median values and interquartile ranges and baseline is presented as 0.01 for better visualization on the log scale.

**Figure 3:**
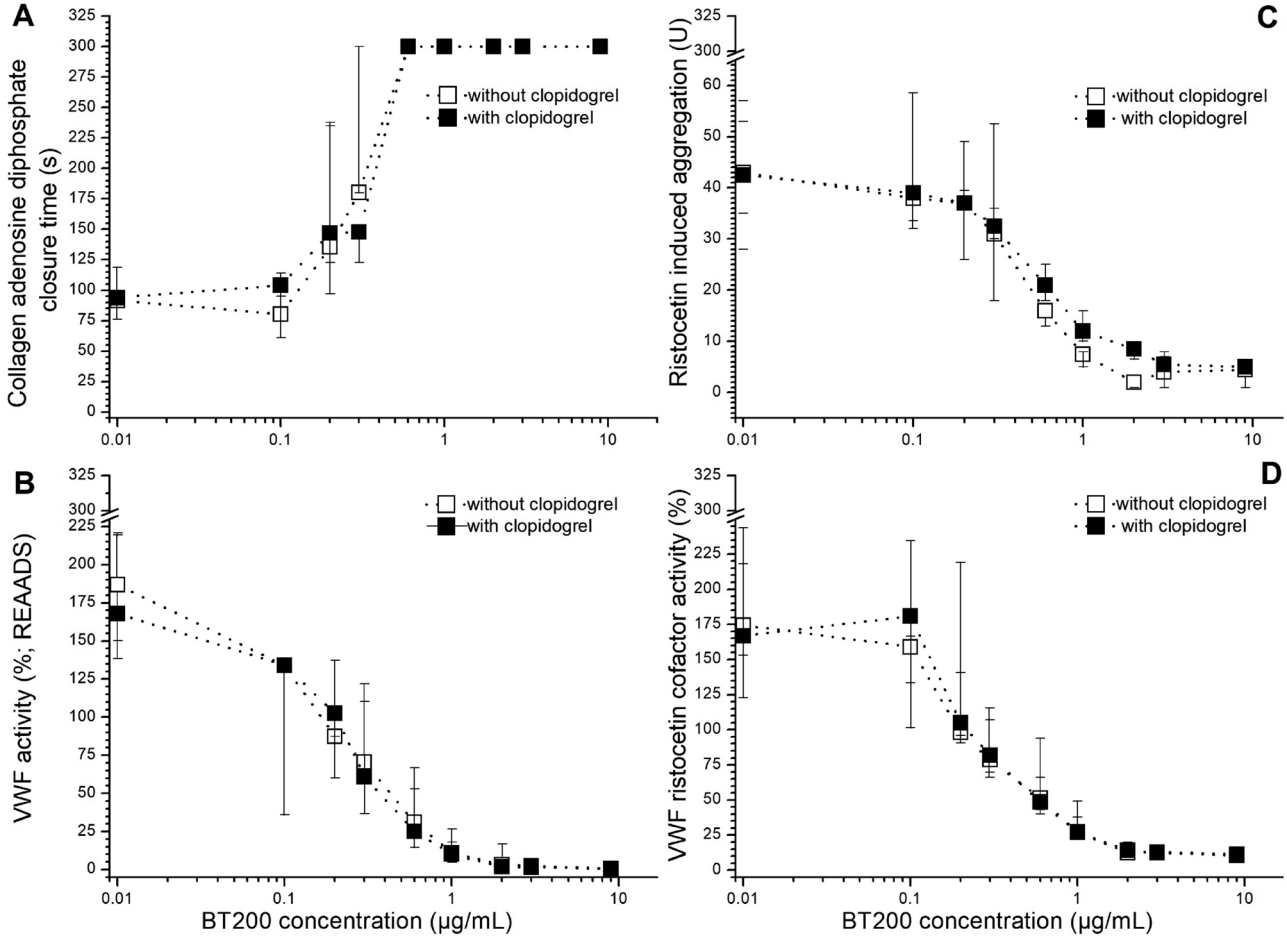
Concentration effect curves of BT200 on platelet plug formation under high shear stress (**A**), ristocetin-induced platelet aggregation (**B**), von Willebrand Factor (VWF) activity (**C**) and ristocetin cofactor activity (VWF:RCo) (**D**) in patients with large artery atherosclerosis stroke or transient ischemic attacks with (n = 18) or without clopidogrel therapy (n = 15). Data are presented as median values and the interquartile range, and baseline is presented as 0.01 for better visualization on the log scale.

In a sensitivity analysis, we stratified patients into two groups: with normal^29^ (median: 150%, range 67–178%; n = 17) or elevated VWF activity (median: 221%, range 184–423%; n = 16). Significantly higher concentrations were needed to lower VWF activity to less than 20% of normal in patients with higher VWF activity levels (Suppl. fig 1), measured with REAADS ELISA, VWF:RiCo (Fig. 2; p < 0.001), or to inhibit VWF dependent platelet function. In an additional sensitivity analysis, we examined the difference between patients who received clopidogrel and those who did not receive it, and no significant difference was noticed in any of the four assays (Fig. 3; p > 0.05).

BT200 did not alter clotting time as measured by thromboelastometry (not shown), indicating no unspecific effect on in vitro coagulation.

## Discussion

Prevention of secondary strokes following an initial stroke is an area that has recently received attention since it is one of the few remaining frontiers in cardiovascular medicine where the existing standard of care is sub-optimal^30^. The complex nature of secondary stroke prevention requires consideration of the type and timing of the stroke. First, stroke prevention must be distinguished from the treatment of acute stroke. Second, the prevention of initial strokes must be distinguished from the prevention of subsequent secondary strokes. Apart from aggressive risk factor management (e.g., treatment of hypertension, hyperlipidemia, and/or diabetes), for the prevention of secondary strokes, the sub-type of a stroke suffered in the initial stroke must be considered in the selection of pharmacotherapy.

Prevention of secondary strokes after initial strokes due to Large Artery Atherosclerosis (LAA) requires antiplatelet therapy. The problem with the available therapeutic options is that the risk/benefit profile of the potent platelet inhibitors, so well- established in the domain of coronary disease, does not translate well into the domain of cerebrovascular disease. Aspirin in combination with clopidogrel reduces the number of recurrent strokes^30^ but does not prevent 75% of recurrent events. Ticagrelor, non-significantly reduced the primary endpoint by only 11% in the SOCRATES trial, a secondary stroke prevention study with > 13,000 stroke/TIA patients^31^, and in the THALES trial the combination of ticagrelor with aspirin decreased the risk of composite of stroke or death within 30 days, but the incidence of disability did not differ^32^. Other potent platelet inhibitors indicated for use in patients with acute coronary syndromes such as prasugrel or vorapaxar carry a black box warning and are contra-indicated in stroke patients because of bleeding risk. Improved secondary stroke prevention requires new approaches to platelet function inhibition other than the n^th^ generation ADP/P2Y12 inhibitors. Thus, there is an unmet medical need for novel anti-thrombotic pharmacotherapy with an improved risk/benefit profile for use in secondary stroke prevention.

The stroke sub-type that we are selectively targeting is LAA. Published medical literature strongly implicates VWF to the pathophysiologic mechanism of LAA stroke, most likely because platelet thrombus formation in the setting of atherosclerotic plaque has an important shear-dependent component that is mediated by VWF. No difference in the inhibitory BT200 concentrations was seen in VWF dependent platelet function tests between the patients with or without clopidogrel. This indicates that clopidogrel is suboptimally effective when the pathogenesis of thrombus formation is driven by increased VWF activity. Additionally, it was shown that VWF inhibition by another anti-VWF aptamer called ARC1779 significantly reduces thromboembolism (a strong predictor of stroke) in patients after carotid endarterectomy^33^ while other antiplatelet drugs including clopidogrel failed to show that effect^34^.

Numerous epidemiologic studies have established that an excess of VWF indicates an increased risk of stroke^35^, and stroke mortality^36^, but even patients with normal VWF levels can have first or recurrent LAA strokes; conversely, a relative deficiency of VWF may be protective^29,37^. Patients with average VWF plasma levels of 24% had a 35–67% reduced risk for ischemic stroke as compared to controls, suggesting that partial inhibition of VWF could be protective in the stroke-prone population^29^.

Findings from this study are essential for planning of trials with VWF inhibitors such as BT200 because our previous experiments did not provide insight into the effects of BT200 on platelet function in any patients with atherosclerotic disease. For the first time, we were able to examine BT200 effects in patients with chronically elevated VWF, which may not be the same as newly released VWF such as seen after desmopressin or endotoxin administration^38^. Freshly released VWF is ultra-large and hyperactive but under normal conditions, it is rapidly degraded by ADAMTS13 which prevents micro thrombosis^39^. There is a marked difference in the levels of VWF and VWF-propeptide in healthy individuals after desmopressin/endotoxin when compared to patients with diabetes, thrombotic thrombocytopenic purpura (TTP), or sepsis^40^. This results from differences in altered secretion but also de novo synthesis of VWF due to enhanced transcriptional activity^40^.

For this reason, it was of interest to investigate the effects of BT200 on platelet function not only in healthy individuals after stimulated VWF release, which we did in a previous study^38^ but also in a patient group with chronically activated endothelium. The patient population in our study had a mean of age of 72 years and different comorbidities, which are all known to enhance VWF levels^41^. In such a population the endothelium is chronically activated and chronic inflammation is present, which is influencing platelet function ^42–44^. Importantly, however, there are also changes in platelet function associated with aging^45,46^, but very limited information is available in an elderly population^47^. This was a relevant shortcoming of our previous study^38^ testing platelet function in healthy volunteers. The reason why we chose this specific subset of stroke patients, is because LAA stroke patients represent the lead target indication for BT200, for obvious reasons which were recently reviewed^35^.

As mentioned, in secondary stroke prevention of LAA stroke, patients are treated either with aspirin, aspirin plus dypiridamole, or clopidogrel^48^ or a combination thereof, while ticagrelor use will likely become more common^32^. Patients in our study were treated with aspirin or clopidogrel, which allowed us to investigate possible interactions of BT200 with those antiplatelet drugs. Clopidogrel and other potent P2Y12 inhibitors such as prasugrel have been proven to inhibit not only platelet activation and aggregation but prasugrel also reduced levels of thromboxane A2^49^ and both prasugrel and clopidogrel reduced platelet interaction with monocytes and leukocytes in mice^49,50^. This is important because platelet-leukocyte aggregates play a major role and modulate the development and progression of vascular diseases^51^. In a study with another anti-VWF aptamer ARC15105, this aptamer reduced the aggregation of platelets induced by a number of different agonists including ristocetin, collagen, adenosine diphosphate, and thrombin receptor activating peptide. This possibly reflects outside/in signalling and this inhibitory effect of ARC15105 was significant both in healthy individuals but also in myocardial infarction patients who were all pretreated with aspirin, clopidogrel, and heparin^52^. Such a possible synergist effect can only be seen in platelet function tests applied to blood of the target population. Therefore, it was of interest to characterize whether the effect of BT200 would be synergistic and/or specific when used in combination with aspirin or clopidogrel. In our study, the intake of other antiplatelet drugs did not influence in vitro effects of BT200.

It is important to mention that some assays such as the VWF ELISA and RiCo tests, which are used to measure VWF activity, usually require high dilution (up to 20-fold) of the samples. High dilution of samples could influence the binding of the aptamer to VWF and can wash the aptamer of its target. This could lead to an overestimation of the doses of BT200 needed for the appropriate VWF inhibition, which may lead to undue interference with the safety of the drug. In contrast platelet function tests performed in fresh blood usually do not require dilution or only minimal dilution, which provides more accurate estimates for concentration-effect curves, and therefore subsequent dosing. In addition, the PFA assay allows measuring VWF function under high shear rates, which is the physiological activator inducing conformational change and therefore activation of VWF. We have noticed that slightly higher concentrations of BT200 are needed for the expected effect when using tests with a high dilution of the samples when compared to the whole blood tests without or with a low dilution of the sample. This is important for estimating the effective dose of BT200 that will be used in clinical trials and it is essential that these estimations are correct. For all of the mentioned reasons, this study delivered important insights for planning future clinical trials with VWF inhibitors.

In this study, we have stratified our population into two groups, patients with VWF activity below or above 180%. Similar to previous studies where we investigated ex vivo effects of BT200 in healthy volunteers after stimulated VWF release with endotoxin or desmopressin^38^ and in acute coronary syndrome (ACS) patients^53^, we have again shown that higher concentrations of BT200 were needed for the same effect in cases of increased VWF when compared to normal VWF levels.

In all three studies of our group, BT200 inhibited VWF in a target-concentration dependent manner. Similarly, another VWF inhibitor called caplacizumab (anti-VWF antibody) was characterized by a target-mediated disposition and the exposure was dependent upon drug and target concentration over time^54^. This suggests that this effect could be a class effect of anti-VWF agents, this finding is important for further insights in pharmacokinetics and effects of BT200.

Finally, it is important to mention that a specific antidote for BT200 has been synthesized and evaluated^55^. Because BT200 is an aptamer, synthesis of an antidote is a rather simple procedure. Inhibition of BT200 in a monkey model has been effective and safe and it is yet to be evaluated in humans. Having a highly specific, effective, and nontoxic antidote is beneficial and it is an advantage especially in cases of any adverse events, such as bleeding or in cases of emergency procedures.

## Conclusion

The anti-VWF aptamer BT200 effectively inhibited VWF activity even in the presence of high VWF levels found in LAA stroke patients, in a target concentration-dependent manner. Together with a long half-life supporting once-weekly administration, and the existence of a reversal agent, BT200 is an interesting drug candidate for secondary prevention of LAA stroke as well as rarer stroke entities with an unmet medical need.

## Limitations

The number of patients included in the study was limited but provides good precision to estimate the variability of effective levels of the VWF inhibitor. Additionally, concentration-effect curves were established ex vivo. Nonetheless, effective concentrations may be directly extrapolated to in vivo application, because BT200 inhibits a plasma protein^10^. So far it has not been determined which are exact levels of VWF inhibition that could prevent arterial thrombosis. In the epidemiologic data, it is shown that patients who suffered from von Willebrand disease with VWF activity of about 25%^29^, had approximately 40–60% fewer arterial thrombotic events, which is why we assumed that decreasing VWF activity to 20% or less is of therapeutic interest.

This study cannot provide estimates about bleeding risks associated with BT200, but von Willebrand disease is characterized by sporadic mucocutaenous bleedings which are not spontaneous except for exaggerated menstrual bleedings, a condition rarely prevalent in LAA stroke patients. Nevertheless, considering the potential for bleeding caused by trauma or surgery, a specific reversal agent for BT200 has been created and successfully tested in nonhuman primates^55^.

## Supplementary Information

Supplementary Information
