# Supplementary material for: The aptamer BT200 blocks von Willebrand factor and platelet function in blood of stroke patients
Source: Sci Rep. 2021 Feb 4;11:3092. doi: 10.1038/s41598-021-82747-7 (PMC7862663; doi:10.1038/s41598-021-82747-7)
Supplement: Supplementary file 1 — Supplementary Information [file 41598_2021_82747_MOESM1_ESM.docx]

**The Aptamer BT200 Blocks Von Willebrand Factor and Platelet Function in Blood of Stroke Patients**

Katarina D. Kovacevic^1^, Stefan Greisenegger^2^, Agnes Langer^2^, Georg Gelbenegger^1^,

Nina Buchtele^3^, Ingrid Pabinger^4^, Karin Petroczi^1^, Shuhao Zhu^5^, James C Gilbert^5^, Bernd Jilma^1^

^1^Department of Clinical Pharmacology,

^2^Department of Neurology,

^3^Department of Internal Medicine I,

^4^Department of Internal Medicine I, Division of Hematology

all at Medical University of Vienna, Vienna, Austria

^5^Guardian Therapeutics, Lexington, MA, USA

**Number of figures:** 3, **Number of tables:** 1, **Supplemental data:** 1

Corresponding author:
Katarina Kovacevic, MSc
Währinger Gürtel 18-20, 1090 Vienna, Austria
Tel.: +43 1 40400 29810
Fax: +43 1 40400 29980
Email: *katarina.kovacevic@meduniwien.ac.at*

Supplementary data


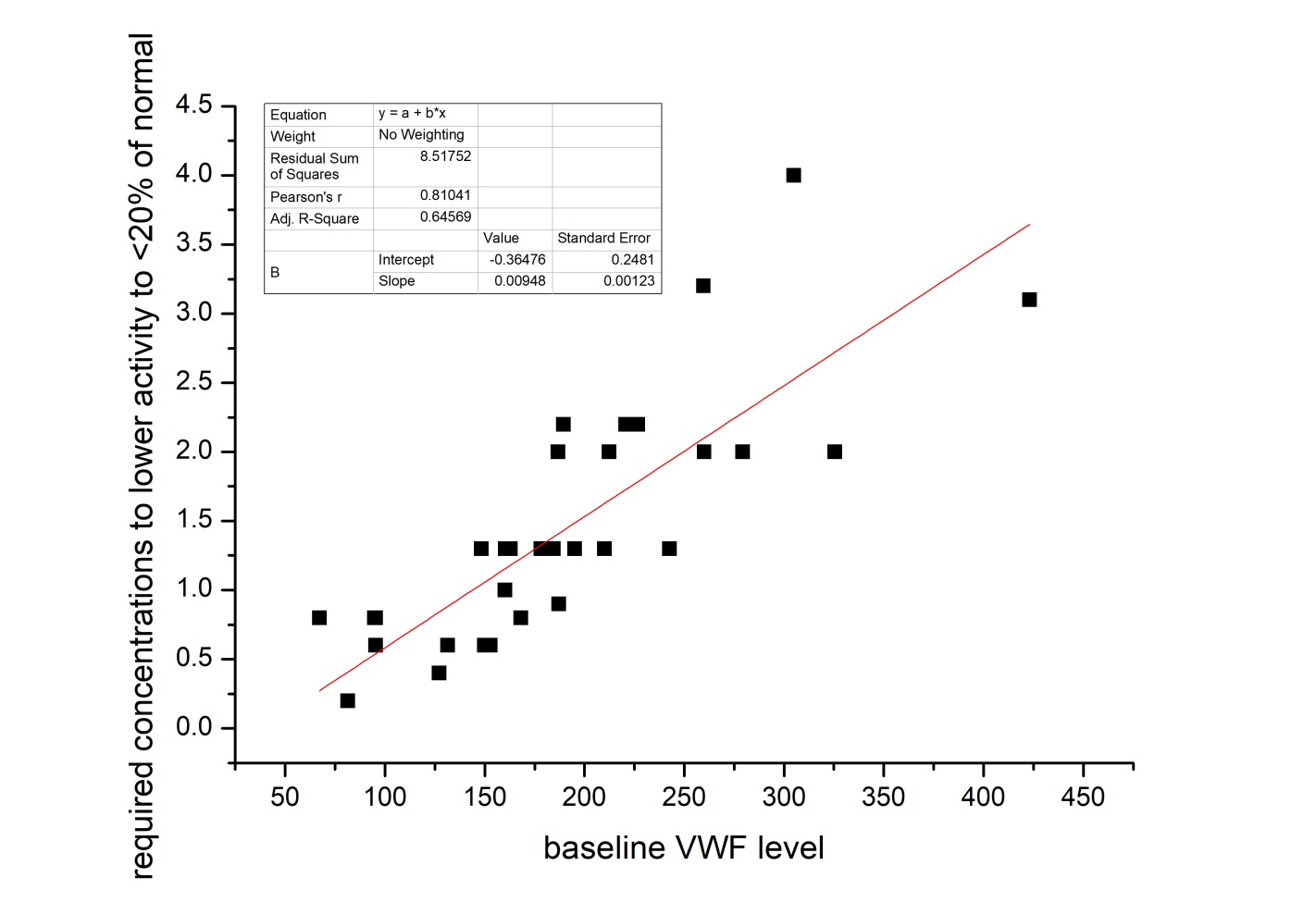


Suppl. Figure 1. Correlation test to estimate the association between baseline VWF levels and BT200 concentrations needed to reduce VWF activity to <20% of normal.
